# Key Amniotic Fluid miRNAs as Promising Target Molecules for the Antenatal Prevention of Pulmonary Hypoplasia Associated with Congenital Diaphragmatic Hernia

**DOI:** 10.3390/ijms26083872

**Published:** 2025-04-19

**Authors:** Angelika V. Timofeeva, Ivan S. Fedorov, Yuri I. Naberezhnev, Nana K. Tetruashvili, Gennady T. Sukhikh

**Affiliations:** National Medical Research Center for Obstetrics, Gynecology and Perinatology Named After Academician Kulakov V.I., 117997 Moscow, Russia; is_fedorov@oparina4.ru (I.S.F.); yu_naberezhnev@oparina4.ru (Y.I.N.); n_tetruashvili@oparina4.ru (N.K.T.); g_sukhikh@oparina4.ru (G.T.S.)

**Keywords:** deep sequencing, polymerase chain reaction (PCR), amniotic fluid, miRNA, congenital diaphragmatic hernia (CDH), lung hypoplasia, pulmonary hypertension

## Abstract

Congenital diaphragmatic hernia (CDH) remains associated with high morbidity and mortality, primarily due to pulmonary hypoplasia and hypertension. Current antenatal diagnostic methods, such as ultrasound and magnetic resonance imaging (MRI), are unable to assess the severity of defects in lung and pulmonary vascular structures, which are critical determinants of the diverse phenotypes of CDH. Aberrant epigenetic regulation of lung development during gestation is believed to play a significant role in the pathogenesis of CDH. In this study, we aimed to identify miRNA patterns in amniotic fluid capable of categorizing CDH-fetuses for the personalized selection of effective treatment strategies at the antenatal and/or postnatal stages. Using deep sequencing and quantitative real-time polymerase chain reaction (PCR), we identified a set of miRNAs—miR-485-3p, miR-320b, miR-320a-3p, miR-221-3p, miR-200b-3p, miR-100-5p, miR-92a-3p, miR-30c-5p, miR-26a-5p, and let-7c-5p—whose reduced expression in amniotic fluid at 19–24 weeks of gestation allowed us to categorize fetuses with CDH into two distinct groups: one significantly different from the control group (non-CDH) and the other closely resembling it. Notably, no significant correlations were found between the content of these miRNAs in amniotic fluid and severity of lung hypoplasia assessed by ultrasound or MRI. However, there was significant positive correlation between the level of each of the miRNAs with that of miR-200b-3p, whose role in ensuring proper bronchopulmonary tissue structure during prenatal development—as well as its therapeutic potential for CDH-associated hypoplastic lungs—has been previously demonstrated. These findings lay the groundwork for the future development of genetically engineered drug formulations designed for antenatal endotracheal administration to correct abnormal miRNA levels in lung tissue and mitigate the progression of pulmonary hypoplasia and hypertension in CDH-fetuses.

## 1. Introduction

Congenital diaphragmatic hernia (CDH) is one of the most complex congenital anomalies in fetuses, characterized by a defect in the diaphragm that allows abdominal organs to move into the thoracic cavity The incidence of CDH is approximately 2–3 cases per 10,000 live births [1]. Despite significant advancements in the diagnosis of CDH and improvements in the resuscitation of newborns with this condition, perinatal and neonatal mortality rates remain alarmingly high, reaching 80% in developing countries and 40–50% in developed ones [2,3]. The primary causes of mortality and severe morbidity in newborns with CDH are lung hypoplasia (LHP) and pulmonary hypertension (PH) [4]. PH associated with CDH manifests as persistently elevated pressure in the pulmonary artery, leading to alterations in pulmonary circulation, impaired gas exchange, multi-organ failure, and ultimately fatal outcomes [5]. However, the mechanisms underlying early lung development in these patients remain poorly understood. Research on animal models using nitrofen has proposed a dual lung injury hypothesis, which explains pulmonary hypoplasia in CDH [6]. This hypothesis involves a primary bilateral lung injury occurring before the closure of the pleuroperitoneal canal, followed by a secondary lung injury due to mechanical compression of thoracic organs resulting from the diaphragmatic defect. The development of pulmonary vessels occurs concurrently with the progressive branching of the bronchial tree [7]. The severity of PH in CDH depends on the extent of disrupted morphogenesis of the lung vascular network and the degree of endothelial cell dysfunction in the pulmonary arteries [8].

An association with chromosomal disorders is observed in 10–30% of cases [9]. In 85% of cases, CDH is left-sided, while in 13% of cases, it is right-sided; other locations are exceedingly rare [10].

The combination of liver herniation and the observed-to-expected contralateral lung-to-head ratio (o/e LHR) measured by ultrasound, or the observed-to-expected total lung volume (o/e TLV) measured by magnetic resonance imaging (MRI), are widely accepted methods for stratifying fetuses with left- and right-sided CDH into groups with varying degrees of pulmonary hypoplasia and corresponding mortality rates [10]. The o/e LHR has been shown to be an independent predictor of postnatal survival in both left- and right-sided CDH, with a sensitivity for survival prediction ranging from 0.49 to 0.98 and a specificity ranging from 0.13 to 0.83 [11]. To improve the accuracy of predicting the severity of LHP and PH, new molecular markers for CDH are needed, which would enable non-invasive decision-making regarding treatment strategies for such patients during the second trimester of pregnancy.

Notably, emerging evidence highlights microRNAs (miRNAs) as key regulators in CDH-associated pulmonary vascular dysfunction. These small noncoding molecules modulate gene expression by suppressing mRNA translation or promoting its degradation [12,13]; they are implicated in pathogenic mechanisms such as endothelial dysfunction and dysregulated signaling in pulmonary arterial smooth muscle cells, driving excessive proliferation and apoptosis resistance [4,14,15,16]. Central to this vascular remodeling are disrupted pathways involving vascular endothelial growth factor, retinoic acid, endothelin, bone morphogenic protein, and apelin.

Changes in miRNA expression patterns in amniotic fluid, tracheal fluid, and lung tissue have also been observed following antenatal treatment for CDH using fetoscopic endoluminal tracheal occlusion (FETO) [17,18,19]. Despite the increased survival of fetuses with severe pulmonary hypoplasia due to CDH after FETO, there is a substantial risk of prelabor rupture of membranes and preterm birth [20,21]. Therefore, there is a pressing need for minimally invasive and highly effective personalized methods to diagnose the severity of LHP in CDH during the antenatal period, allowing for reliable prognosis and the selection of appropriate treatment strategies.

We proposed a novel screening strategy based on the quantification of specific miRNAs in amniotic fluid as markers of LHP and PH associated with CDH at 19–24 weeks of gestation (GW). The information obtained can be used to assess disease severity during fetoscopy and amniotic fluid sampling, as well as for subsequent targeted therapy aimed at correcting altered miRNAs through antenatal endotracheal administration of genetically engineered constructs to mitigate the severity of CDH.

## 2. Results

### 2.1. Predictive Value of Instrumental Tools in Assessing Survival of Newborns with Congenital Diaphragmatic Hernia (CDH)

The study group (Section 4.1) was stratified into subgroups based on the severity of pulmonary hypoplasia in fetuses, as determined by the observed/expected lung-to-head ratio (o/e LHR), cardiac compression index (CCI, calculated as the ratio of heart length to its thickness), and observed/expected total fetal lung volume (o/e TFLV) measured at 19–24 weeks of gestation. These subgroups—mild, moderate, and severe—were further analyzed for gestational age at delivery, neonatal weight, and perinatal mortality rates (Table S1). To evaluate the predictive power of o/e LHR, CCI, and o/e TFLV in estimating the survival probability of fetuses with CDH, the analysis focused exclusively on pregnancy cases from two distinct cohorts: (1) pregnancies where newborns demonstrated positive clinical progress throughout NICU treatment until discharge (37 cases), and (2) pregnancies where newborns died prior to undergoing surgical intervention for CDH (27 cases). Cases involving pregnancy termination due to extreme pulmonary hypoplasia or neonatal death following surgical treatment were excluded from the analysis.

**Figure 1:**
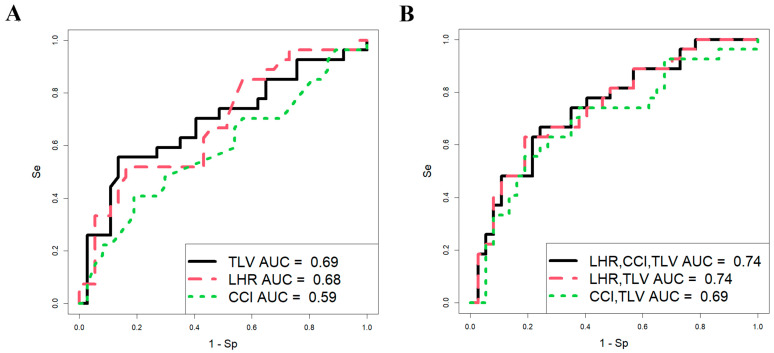
Predictive significance of ultrasound and MRI data in assessing the survival of fetuses with CDH. Logistic regression models constructed for each parameter (**A**) and their combination (**B**).

Using RStudio software (version 1.4.1106, accessed on 25 January 2025), a stepwise inclusion and exclusion approach was employed to assess the contribution of each parameter (o/e LHR, CCI, and o/e TFLV) in development of logistic regression models. The analysis was conducted on a sample of 64 CDH patients (Figure 1), with neonatal survival during the early neonatal period serving as the dependent variable (0 = alive, 1 = fatal outcome).

The parameters of the models in Figure 1 are presented in Table 1, which shows that the predictive ability of o/e LHR, CCI, and o/e TFLV in assessing fetal survival in the neonatal period individually has statistical significance (models 1 and 2, Table 1; Figure 1A), except for CCI (model 3, Table 1; Figure 1A). However, these parameters are characterized by low sensitivity (51.9% for o/e LHR and 55.6% for o/e TLV), meaning that the probability of correctly predicting truly positive outcomes (newborns with a fatal outcome in the neonatal period) is approximately 50%, with an equivalent likelihood of false-positive predictions. This could lead to inappropriate management strategies for the pregnant woman, such as either termination of pregnancy or FETO, which carries a high risk of preterm birth and associated neonatal complications.

When using various combinations of o/e LHR, CCI, and o/e TFLV in development of logistic regression models (Figure 1B, Table 1), the most statistically significant combination was o/e LHR and o/e TFLV (model 5, Table 1). However, the proportion of correctly predicted negative outcomes (i.e., newborns who would survive) was only 63%, while the proportion of false-negative outcomes was 37%, indicating a significant risk of missing severe cases of CDH that result in death. Thus, the models based on o/e LHR, CCI, and o/e TFLV in our sample set demonstrated low predictive ability in assessing the severity of CDH in fetuses, making it challenging to determine appropriate management strategies until delivery. Consequently, we sought to identify other markers of fetal severity in CDH during the antenatal period.

### 2.2. Identification of miRNA Markers in CDH

**Figure 2:**
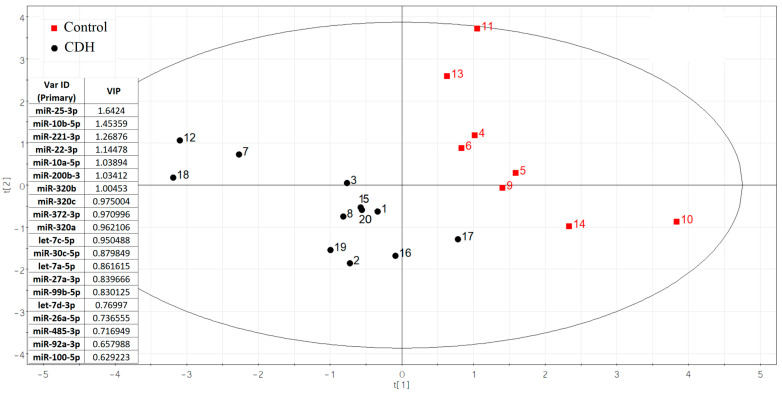
The partial least squares (PLS) regression analysis of miRNA sequencing data in amniotic fluid samples from fetuses with and without CDH.

Using small RNA deep sequencing of amniotic fluid from 20 pregnancies (12 CDH cases [Table S1, Sheet 1]; 8 controls [Table S1, Sheet 2]) at 19–24 weeks, we identified 108 miRNAs per group after applying an RStudio-based filtering algorithm to exclude miRNAs with fewer than 10 reads in ≥65% of samples. This approach preserved group-specific miRNAs while eliminating unstable or uninformative candidates. From these results, 75 miRNAs with ≥10 reads per sample were analyzed for differential expression via negative binomial modeling (DESeq2). Ultimately, 20 miRNAs showed the strongest discriminatory power between CDH and control samples (Figure 2).

**Figure 3:**
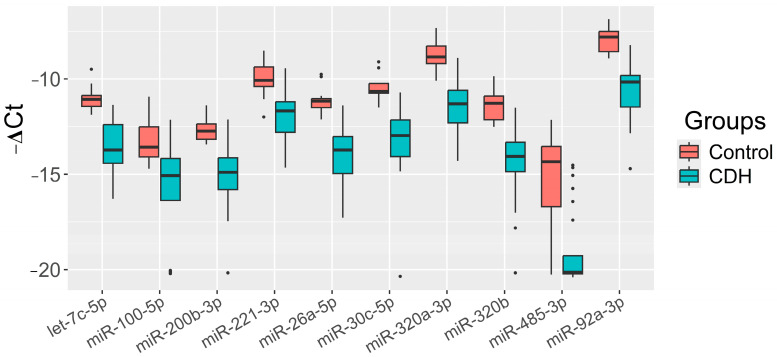
Levels of miRNAs (“−ΔCt” values) in the amniotic fluid of fetuses with CDH relative to those without CDH (control).

The significance of the 20 miRNAs listed in the insert of Figure 2 and associated with CDH was validated by quantitative RT-PCR on a sample set of 56 patients (33 controls, 23 CDH, Table S1). The relative levels of miRNAs in the amniotic fluid were determined using the ∆Ct method, with UniSp6 as the reference RNA. Statistically significant differences (*p* < 0.05) between the two groups were found for let-7c-5p, miR-100-5p, miR-26a-5p, miR-30c-5p, miR-320a-3p, miR-320b, miR-92a-3p, miR-200b-3p, miR-221-3p, and miR-485-3p (Table 2). A box plot was constructed to visualize the data, showing that the levels of these 10 miRNAs were significantly reduced in the amniotic fluid of patients with CDH compared to controls (Figure 3).

**Figure 4:**
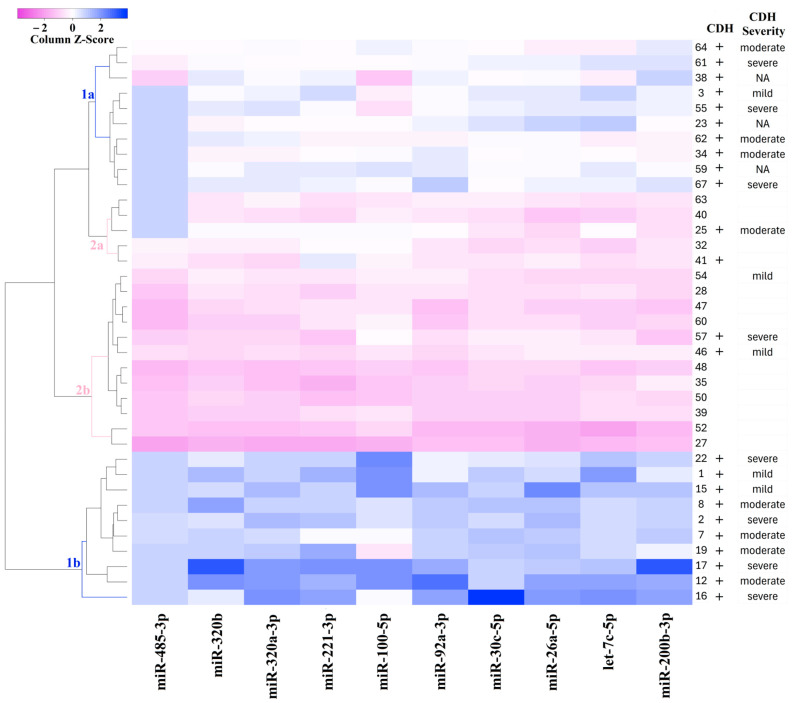
Heatmap of the amniotic fluid samples from fetuses with or without CDH based on the “−∆Ct” values of miRNAs.

For the classification of amniotic fluid samples based on quantitative PCR data, a heatmap was constructed for the levels of miR-485-3p, miR-320b, miR-320a-3p, miR-221-3p, miR-200b-3p, miR-100-5p, miR-92a-3p, miR-30c-5p, miR-26a-5p, and let-7c-5p (Figure 4). The heatmap uses a color gradient, with deep blue indicating the most reduced levels of miRNAs and deep pink indicating the most elevated levels. Figure 4 clearly shows the formation of four sample clusters: CDH (clusters 1a and 1b) and non-CDH (clusters 2a and 2b). Cluster 1a is grouped with clusters 2a and 2b due to similar miRNA expression profiles, whereas cluster 1b is distinctly separated from the other clusters. Since cluster 1b exhibited the most significant reduction in miRNA levels relative to the control group compared to cluster 1a, which occupies an intermediate position between cluster 1b and clusters 2a and 2b, it can be inferred that cluster 1b represents more pronounced structural changes in the bronchopulmonary tissue and vascular system of the lungs in CDH patients.

In Figure 4, no clear relationship is observed between the severity of CDH, as determined by o/e LHR, CCI, and o/e TFLV (as described in the Materials and Methods section), as well as the miRNA profile in each cluster. This suggests that the severity of the fetal condition in CDH may be determined not only by the overall reduction in lung tissue volume, as measured by instrumental methods, but also by structural changes in the tissue, potentially linked to imbalances in miRNA expression during embryonic development.

**Figure 5:**
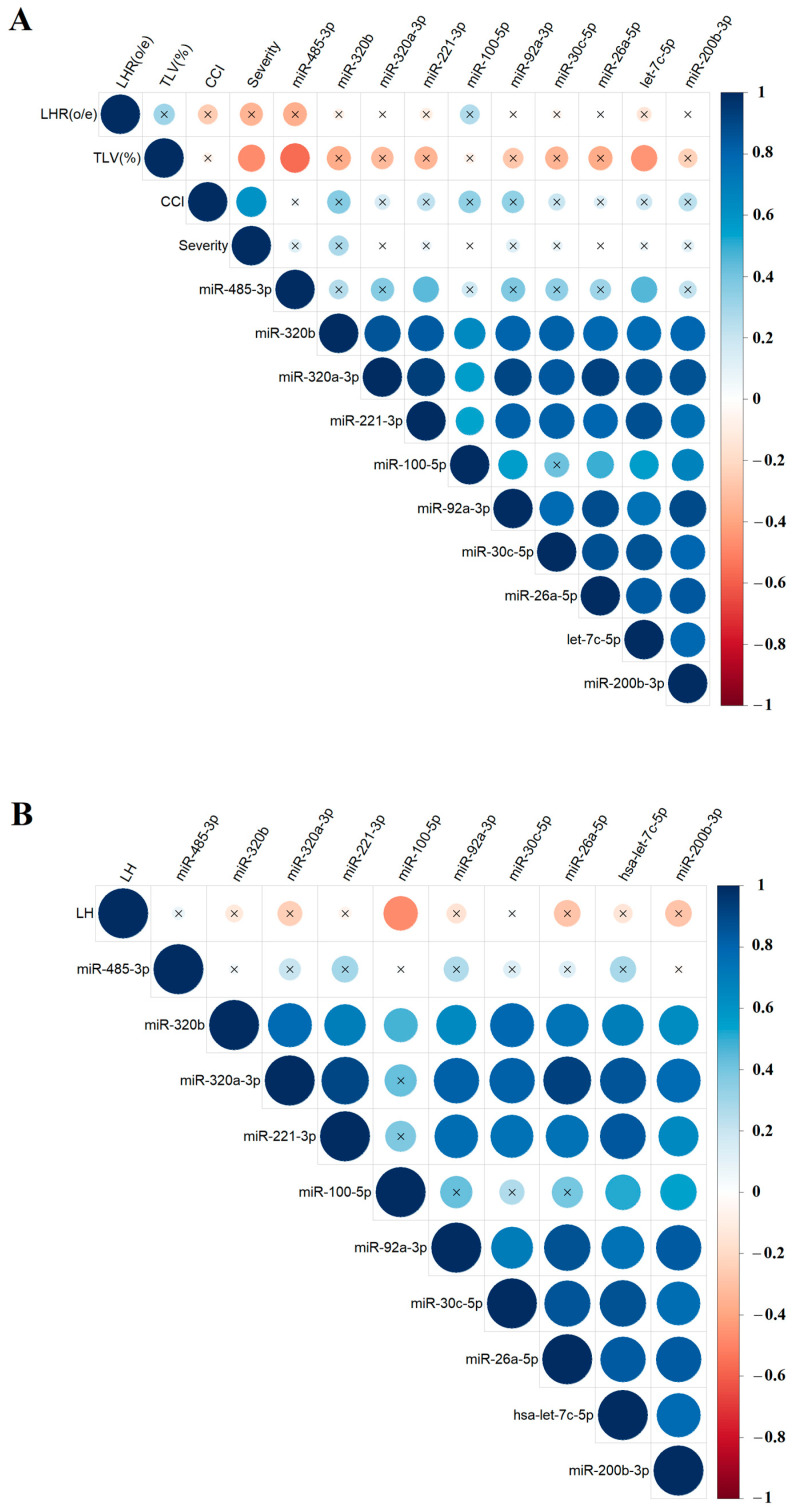
Correlation matrix based on the analysis of miRNAs and instrumental parameters of fetal examination in the CDH group, obtained using the nonparametric Spearman rank correlation method. Correlations of the “−∆Ct” values for specific miRNAs with the values of o/e LHR, CCI, and o/e TFLV (**A**), as well as the %LH values (**B**). Positive and negative correlations are indicated in blue and red, respectively. Significant correlations (*p* < 0.05) are marked with a dot, while nonsignificant correlations are marked with a cross. O/eLHR—observed/expected lung-to-head ratio; o/eTLV—observed/expected total fetal lung volume; %LH—liver herniation percentage; severity—the severity of PH as determined by ultrasound and MRI.

To explore potential relationships between the levels of specific miRNAs in amniotic fluid and the results of clinical and instrumental fetal examinations, the nonparametric Spearman’s rank correlation method was used. Correlations between miRNA levels and the values of o/e LHR, CCI, o/e TFLV (Figure 5A), and the %LH value (Figure 5B), calculated as the ratio of the hepatic volume above the diaphragm to the total liver volume, were presented as separate matrices, as %LH values were not available in ultrasound reports for all fetuses. The study identified several significant correlations: hsa-miR-485-3p and hsa-let-7c-5p showed inverse correlations with o/e TLV (r = −0.57, *p* = 0.007 and r = −0.45, *p* = 0.0349, respectively; Figure 5A, Table S2, Sheet 1 and 2), while hsa-miR-100-5p showed a inverse correlation with %LH (r = −0.48, *p* = 0.0455, Figure 5B, Table S2, Sheet 3 and 4). No significant correlations were found between miRNA expression levels in amniotic fluid and the severity of PH as determined by ultrasound and MRI. However, most miRNAs involved in the construction of the correlation matrices in Figure 4 showed statistically significant positive correlations with each other, indicating a pattern of miRNAs with similar expression changes associated with CDH, which could be used to develop targeted antenatal therapies for LHP and PH in fetuses.

**Figure 6:**
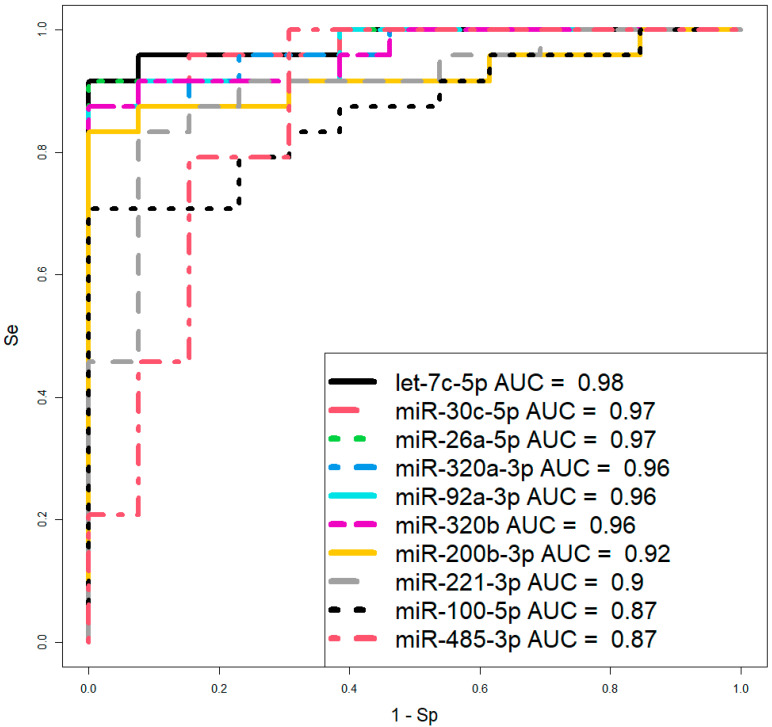
ROC curves of logistic regression models based on real-time PCR data comparing the CDH group and control group (without CDH) for the content of miRNA in amniotic fluid at 19–24 weeks of pregnancy. Se—sensitivity; Sp—specificity.

However, before proceeding to therapeutic interventions to confirm the diagnosis of CDH in the fetus, established by ultrasound and MRI at 19–24 weeks of gestation, we propose using two logistic regression models based on the levels of let-7c-5p and miR-26a-5p (Figure 6, Table 3) in amniotic fluid, where the dependent variable (response variable) was the diagnosis of the fetus (0—absence of CDH; 1—presence of CDH). These two models were selected over others due to their highest AUC values of 0.98 and 0.97, respectively, the statistical significance of their parameters (Table 3), and their highest sensitivity (91.67%) and specificity (100%).

### 2.3. Functional Significance of Antenatal miRNA Markers in CDH

**Figure 7:**
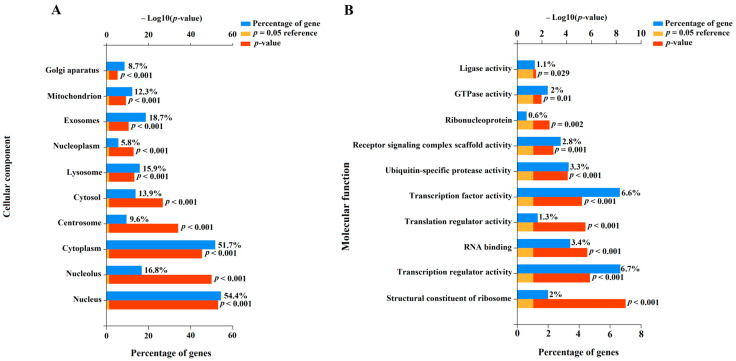
Enrichment analysis of gene targets of miRNA markers of CDH using the FunRich software tool (Version 3.1.3, accessed on 25 January 2025). Histograms were constructed by analyzing protein products of target genes in terms of localization in the cellular component (**A**) and their molecular function (**B**).

To understand the potential role of miR-485-3p, miR-320b, miR-320a-3p, miR-221-3p, miR-200b-3p, miR-100-5p, miR-92a-3p, miR-30c-5p, miR-26a-5p, and let-7c-5p in the pathogenesis of CDH as well as associated LHP and PH, we identified their experimentally validated target genes using the miRTargetLink 2.0 program (Table S3). This was followed by the functional enrichment analysis of the identified gene sets using the FunRich software tool (Version 3.1.3), with a significance threshold of *p* < 0.05 (Figure 7, Table S4).

In terms of cellular components (Figure 7A), 54.4% (*p* < 0.001) of the protein products of the target genes of the aforementioned miRNAs were located in the nucleus, and 51.7% (*p* < 0.001) were found in the cytoplasm. Additionally, 8.7–18.7% of the targets were localized in the Golgi apparatus, centrosome, mitochondrion, lysosome, and exosomes (Figure 7A), indicating their involvement in secretory processes, synthesis and modification of carbohydrates and glycoproteins, intracellular degradation of substances and organelles, energy-dependent processes, apoptosis, cell division, and intercellular communication. Importantly, the protein products of the experimentally validated target genes of the miRNA markers identified in this study are involved in transcription, translation, formation of receptor signaling complex scaffolds, clustering and organization of receptors with intracellular signaling cascades, and ubiquitination processes (Figure 7B). These processes are critical for maintaining cellular homeostasis. Local disruption of the miRNA expression profile and their target genes can lead to structural and functional changes in the corresponding tissues and organs. In this context, the altered expression profile of miR-485-3p, miR-320b, miR-320a-3p, miR-221-3p, miR-200b-3p, miR-100-5p, miR-92a-3p, miR-30c-5p, miR-26a-5p, and let-7c-5p in the amniotic fluid of fetuses with CDH may reflect pulmonary hypoplasia and structural changes in the lung vascular system.

## 3. Discussion

In this study, we analyzed the predictive significance of instrumental methods in assessing fetal survival in the early neonatal period. Based on our findings, we concluded that the existing predictive indices for CDH at 19–24 weeks of gestation—o/e LHR, CCI, and o/e TFLV—lack high reliability. Even when combining o/e LHR and o/e TFLV in logistic regression models, the proportion of correctly predicted negative outcomes (i.e., newborns who would survive) was only 63%, while the proportion of false-negative outcomes was 37%, indicating a significant risk of missing severe cases of CDH that result in death. This may be due to the fact that the fatal outcome of the newborn may be predetermined by factors established at an earlier stage of embryogenesis. Given that cases with genetic abnormalities were excluded from our study, it was logical to search for pathological changes at the molecular level. In this regard, invasive prenatal diagnosis by amniocentesis to analyze the content of miRNAs as representatives of small non-coding RNAs with regulatory functions in cellular signaling pathways was deemed appropriate.

Using deep sequencing followed by validation with quantitative real-time PCR, we identified a miRNA profile associated with CDH, characterized by statistically significant reductions in the levels of miR-485-3p, miR-320b, miR-320a-3p, miR-221-3p, miR-200b-3p, miR-100-5p, miR-92a-3p, miR-30c-5p, miR-26a-5p, and let-7c-5p in the amniotic fluid of fetuses at 19–24 weeks of gestation compared to controls. Notably, only a few miRNAs showed significant correlations with instrumental parameters: hsa-miR-485-3p and hsa-let-7c-5p were inversely correlated with o/e TLV, while hsa-miR-100-5p was inversely correlated with %LH. This suggests that the biological significance of changes in molecular markers and instrumental data differ while comparing CDH patients with controls. Furthermore, we identified two subgroups within the CDH group: one subgroup exhibited a miRNA profile with reduced levels but close to the reference (control without CDH), while the other subgroup showed a markedly reduced expression of these miRNAs compared to the control group. Bioinformatics analysis of the functions of the protein products of the experimentally validated target genes of the miRNA markers identified in this study revealed their involvement in transcription, translation, intracellular signaling, and ubiquitination processes. These processes are critical for maintaining cellular homeostasis, and their dysregulation during early embryogenesis can lead to impaired organ and tissue development, including structural abnormalities in the lungs and pulmonary vasculature.

Animal model studies using nitrofen have proposed a dual lung injury hypothesis, which includes a primary bilateral lung injury occurring before the closure of the pleuroperitoneal canal and a secondary lung injury due to mechanical compression of thoracic organs resulting from the diaphragmatic defect [6]. Aberrant pulmonary vascular development in CDH occurs in parallel with airway branching and takes place between 4 and 16 weeks of gestation [14]. The severity of PH in CDH depends on the degree of impaired morphogenesis of the lung vascular network and the dysfunction of endothelial cells in the pulmonary arteries [22]. These structural changes in the bronchopulmonary and vascular systems cannot be fully captured by the aforementioned instrumental methods. This is further supported by the lack of correlation between the severity of CDH, as determined by o/e LHR, CCI, and o/e TFLV, as well as the miRNA profile, as demonstrated by the Spearman correlation analysis in this study. It is possible that the severity of the fetal condition in CDH is determined not only by the overall reduction in lung tissue volume, as measured by instrumental methods, but also by structural changes in the tissue, potentially linked to imbalances in miRNA expression during embryonic development.

The involvement of the miRNA markers identified in this study in the pathogenesis of various pulmonary diseases has been already published. For example, reduced expression of miR-26a-5p [23], miR-92a-3p from the polycistronic miR-17–92 cluster [24,25], and miR-320a-3p [26] have been implicated in the development and progression of pulmonary fibrosis. In cases of PH, reduced expression of miR-100-5p and miR-30c-5p [27], miR-17-92 [28], and miR-26a-5p [29] has been observed in human pulmonary artery smooth muscle cells. Downregulation of miR-26a-5p has been reported in lung biopsies of COVID-19 patients, reflecting severe lung injury [30]. The progression of SARS-CoV-2 infection in patients with severe respiratory failure has been associated with a significant decrease in the levels of miR-320a, miR-320b, and miR-320c in peripheral blood. These miRNAs are involved in regulating genes within the Hippo signaling and TGF-β signaling pathways, adherens junctions, and epithelial paracellular permeability [31]. Additionally, plasma levels of miR-320 family members were negatively correlated with CRP, D-dimer, and IL-6 concentrations, highlighting their role in the pathogenesis of inflammatory lung diseases and coagulation processes. In patients with chronic obstructive pulmonary disease (COPD), reduced expression of miR-485-3p [32], miR-221-3p [33,34], miR-320b [35,36], and let-7c-5p [37,38,39] has been observed in pulmonary arteries and correlated with the severity of airflow obstruction. In mouse models of nicotine-induced lung injury, downregulation of let-7c-5p and upregulation of its downstream effector genes encoding pro-fibrotic factors (FN1, COL1A1, and TGF-β1) and proinflammatory factors (IL-6 and IL-1β) have been reported [40].

It is noteworthy that the miRNA markers identified in this study, associated with LHP and PH in CDH-patients, exhibit a specific expression pattern similar to that of miR-200b-3p, as demonstrated by high positive correlations using the nonparametric Spearman method. The importance of miR-200b-3p in ensuring the proper structure of bronchopulmonary tissue during prenatal development has been well-established [14]. In a mouse model with a knockout of the miR-200b gene, significant changes in the distal bronchial tree and alveolar wall structure, suppression of alveolar cell differentiation, reduced surfactant synthesis, and pronounced lung fibrosis were observed [41]. The therapeutic potential of miR-200b-3p has been demonstrated in studies using the nitrofen model of CDH in rats, where intravenous administration of miRIDIAN miR-200b mimics (GE Dharmacon, Mississauga, ON, Canada) at 5 mg/kg prevented the progression of pulmonary hypoplasia [42]. Therefore, the additional miRNAs identified in this study, with expression profiles similar to miR-200b-3p in the amniotic fluid of CDH patients, are considered promising candidates for the development of novel lung-specific therapeutic approaches.

The potential efficacy of these miRNAs in targeting lung tissue is supported by the literature data. For example, the aberrantly low levels of miR-100-5p and miR-30c-5p, which are involved in the pathogenesis of PH, can be normalized using neural stem cell line-derived extracellular vesicles, which contain these miRNAs and exhibit transendothelial permeability into smooth muscle cells [27]. miR-30c-5p has been shown to prevent epithelial–mesenchymal transition (EMT) in lung epithelial cells by targeting connective tissue growth factor (CTGF) and ATG5-associated autophagy [43]. Overexpression of miR-221-3p has been shown to alleviate cell apoptosis and inflammation by targeting CDKN1B in an in vitro model of COPD [33]. Overexpression of miR-320a-3p has been shown to suppress TGFβ-1-induced EMT in alveolar epithelial cells, preventing their conversion into mesenchymal cells and reducing collagen deposition, lung tissue destruction, and pulmonary fibrosis [26]. Overexpression of miR-26a-5p has been shown to alleviate acute lung injury by targeting TLR4 [44] or CTGF [45], thereby inhibiting lung inflammation and cell apoptosis.

Although this study identified a specific miRNA pattern in the amniotic fluid of fetuses with CDH—whose reduced expression levels may reflect the severity of LHP, given their established role in various pulmonary diseases—a key limitation is the absence of an analysis correlating these miRNA levels in the lung tissue of deceased CDH fetuses with the degree of LHP based on histological findings. If such a correlation is confirmed (a focus of our ongoing research), future work should aim to develop genetic constructs encoding miR-485-3p, miR-320b, miR-320a-3p, miR-221-3p, miR-200b-3p, miR-100-5p, miR-92a-3p, miR-30c-5p, miR-26a-5p, and let-7c-5p for endotracheal delivery as a potential therapeutic strategy to mitigate LHP and PH progression in CDH. The dosage and administration frequency of these constructs—whether antenatal or postnatal—would then depend on CDH severity, which could be diagnosed prenatally via the marker miRNAs in amniotic fluid identified here at 19 weeks of gestation.

A clinically actionable outcome of this study is the development of logistic regression models using amniotic fluid levels of let-7c-5p and miR-26a-5p to verify CDH diagnosis, initially detected by ultrasound and MRI at 19–24 weeks.

## 4. Materials and Methods

### 4.1. Patients

The study was conducted at the Federal State Budgetary Institution “National Medical Research Center for Obstetrics, Gynecology, and Perinatology named after Academician V.I. Kulakov” of the Ministry of Health of the Russian Federation between 2021 and 2024. A total of 85 pregnant women whose fetuses were diagnosed with CDH by ultrasound and/or MRI were included in the study (Table S1). The severity of CDH was assessed using a combination of ultrasound parameters: observed/expected lung-to-head ratio (o/e LHR %), liver herniation percentage (LH %), and cardiac compression index (CCI), as well as MRI data: observed/expected total fetal lung volume (o/eTFLV %) [46,47]. The control group consisted of 33 pregnant women who underwent amniocentesis at 19–24 weeks of gestation for medical indications.

Inclusion criteria for the study were pregnant women aged 18 to 45 years, singleton pregnancy, absence of contraindications for invasive prenatal diagnosis, and signed informed consent. Exclusion criteria were threatened preterm labor, preeclampsia, anatomical factors preventing pregnancy continuation, fetal aneuploidy, known structural genomic variants, other serious fetal anomalies or genetic syndromes, and multiple fetal malformations as determined by ultrasound or MRI.

For karyotyping and the detection of pathogenic microdeletions larger than 1000 kbp and microduplications larger than 2000 kbp, invasive prenatal diagnosis by amniocentesis was performed. After local anesthesia with 2% lidocaine, the amniotic cavity was punctured with an 18 G needle and 40 mL of amniotic fluid was collected. Genetic testing of the amniotic fluid was performed using prenatal molecular karyotyping on DNA microarrays (CytoScan, Affymetrix, Santa Clara, CA, USA). Simultaneously, amniotic fluid was collected for the analysis of miRNA composition.

### 4.2. Isolation of RNA from Amniotic Fluid Samples

Amniotic fluid samples were collected in Eppendorf tubes (Hamburg, Germany) and centrifuged for 20 min at 300× *g* at 4 °C. The cell-free supernatant was then collected and centrifuged again for 10 min at 16,000× *g*. RNA was isolated from 200 μL of the cell-free amniotic fluid using the miRNeasy Serum/Plasma Kit (Qiagen, Hilden, Germany).

### 4.3. Deep Sequencing of miRNA

cDNA libraries were synthesized from 6 μL of total RNA eluate using the NEBNext^®^ Multiplex Small RNA Library Prep Set for Illumina^®^ (Set1, New England Biolabs^®^, Frankfurt am Main, Germany, cat. No. E7580S), following the manufacturer’s protocol. The cDNA libraries were amplified for 21 cycles and purified using 6% polyacrylamide gel, with the 140–160 base pair fraction extracted. The quantity and quality of the cDNA libraries were assessed using an Agilent 2100 Bioanalyzer (Agilent Technologies, Waldbronn, Germany) with the High Sensitivity DNA reagents kit (Agilent Technologies, Santa Clara, CA, USA). Sequencing was performed on the NextSeq 500 platform (Illumina, San Diego, CA, USA, cat. no. SY-415-1001) according to the manufacturer’s instructions. Sequence annotation was performed using the GRCh38.p15 and miRBase v21 databases, along with the STAR RNAseq aligner program. The DESeq2 software package (version 1.42.0) was used to normalize the cDNA read counts in each sample.

### 4.4. Reverse Transcription and Quantitative Real-Time PCR

For cDNA synthesis, 5 µL of the 14 µL RNA eluate obtained from the miRNeasy Serum/Plasma Kit column (Qiagen, Hilden, Germany) was used with the miRCURY LNA RT Kit (Qiagen, Hilden, Germany), following the manufacturer’s protocol. Quantitative real-time PCR was performed using the miRCURY LNA SYBR Green PCR Kit (Qiagen, Hilden, Germany) and miRNA-specific primers (miRCURY LNA miRNA PCR Assay, Qiagen, Hilden, Germany) on a StepOnePlus™ thermal cycler (Applied Biosystems, Waltham, MA, USA). Relative miRNA expression was calculated using the ∆Ct method, with UniSp6 as the reference RNA.

### 4.5. Statistical Data Processing

Statistical analysis was performed using scripts written in R 4.3.2 [48] and RStudio software, version 2023.09.1 [49]. The Shapiro–Wilk test was used to assess data normality. For non-normally distributed data, paired comparisons were made using the Mann–Whitney test. Variables that did not follow a normal distribution were described as the median (Me) and quartiles Q1 and Q3 in the format Me (Q1; Q3). A significance threshold of *p* = 0.05 was set for pairwise comparisons, and *p*-values less than 0.001 were indicated as *p* < 0.001.

Two-dimensional hierarchical clustering of real-time RT-PCR data was performed in RStudio using the complete linkage clustering method, with Manhattan distance used to calculate differences between dendrogram nodes.

Logistic regression models were developed in RStudio through stepwise inclusion and exclusion of miRNA markers based on their contribution to the model. The predictive performance of the models was evaluated using ROC (Receiver Operating Characteristic) analysis, with assessment of the AUC (Area Under the Curve), statistical significance, specificity, and sensitivity.

## Figures and Tables

**Table 1:** Characteristics of the logistic regression models in Figure 1A,B.

Parameter	Coefficients	Wald	*p*-Value	Threshold	Sensitivity	Specificity
model 1						
(Intercept)	1.764	1.904	0.057	0.511	0.556	0.865
o/e TLV	−5.070	−2.309	0.021			
model 2						
(Intercept)	1.793	2.108	0.035	0.496	0.519	0.838
o/e LHR	−4.155	−2.535	0.011			
model 3						
(Intercept)	−1.317	−0.784	0.433	0.430	0.407	0.811
CCI	0.760	0.603	0.546			
model 4						
(Intercept)	−2.886	−1.292	0.196	0.569	0.757	0.667
o/e LHR	3.613	2.157	0.031			
CCI	−0.302	−0.229	0.819			
o/e TLV	4.249	1.833	0.067			
model 5						
(Intercept)	−3.311	−2.643	0.008	0.542	0.811	0.630
o/e LHR	3.664	2.206	0.027			
o/e TLV	4.242	1.828	0.058			
model 6						
(Intercept)	−0.766	−0.390	0.697	0.518	0.811	0.556
CCI	−0.752	−0.569	0.569			
o/e TLV	5.056	2.306	0.021			

**Table 2:** Comparison of groups “Control” and “CDH” by the miRNA “−ΔCt” value plotted as a box diagram in Figure 3.

miRNAs	Groups	Me(Q1;Q3), “−ΔCt” Value	Wilcoxon–Mann–Whitney U Test, *p*-Value, Control vs. CDH
hsa-let-7c-5p	Control	−11.07 (−11.43;−10.87)	<0.001
	CDH	−13.72 (−14.42;−12.39)	
miR-100-5p	Control	−13.58 (−14.09;−12.52)	<0.001
	CDH	−15.07 (−16.37;−14.17)	
miR-200b-3p	Control	−12.74 (−13.16;−12.36)	<0.001
	CDH	−14.9 (−15.8;−14.13)	
miR-221-3p	Control	−10.07 (−10.39;−9.37)	<0.001
	CDH	−11.68 (−12.79;−11.2)	
miR-26a-5p	Control	−11.16 (−11.5;−11.04)	<0.001
	CDH	−13.72 (−14.97;−13.03)	
miR-30c-5p	Control	−10.66 (−10.75;−10.24)	<0.001
	CDH	−12.97 (−14.07;−12.15)	
miR-320a-3p	Control	−8.84 (−9.19;−8.27)	<0.001
	CDH	−11.3 (−12.3;−10.6)	
miR-320b	Control	−11.28 (−12.14;−10.89)	<0.001
	CDH	−14.07 (−14.86;−13.32)	
miR-485-3p	Control	−14.34 (−16.7;−13.54)	<0.001
	CDH	−20.14 (−20.22;−19.26)	
miR-92a-3p	Control	−7.8 (−8.56;−7.51)	<0.001
	CDH	−10.16 (−11.47;−9.82)	

**Table 3:** Parameters of the logistic regression models in Figure 6.

Variables	Wald	*p*-Value	Coefficients	Threshold	Se	Sp
Model 1						
(Intercept)	−2.296182879	0.021665427	−65.697	0.7751	0.9167	1
let-7c-5p	2.288968927	0.022081158	5.611			
Model 2						
(Intercept)	−2.462190859	0.013809116	−40.639	0.8585	0.8333	1
miR-30c-5p	2.437656437	0.014782816	3.63			
Model 3						
(Intercept)	−2.553176495	0.010674539	−38.729	0.6989	0.9167	1
miR-26a-5p	2.533123567	0.011305109	3.259			
Model 4						
(Intercept)	−2.891983619	0.003828179	−24.658	0.8026	0.875	1
miR-320a-3p	2.88991503	0.00385346	2.56			
Model 5						
(Intercept)	−2.624764522	0.008670891	−27.35	0.7533	0.875	1
miR-92a-3p	2.618997485	0.00881886	3.126			
Model 6						
(Intercept)	−2.808514607	0.004977062	−29.089	0.7235	0.875	1
miR-320b	2.81443525	0.004886302	2.386			
Model 7						
(Intercept)	−2.858228619	0.004260133	−27.691	0.66	0.8333	1
miR-200b-3p	2.856155607	0.004288049	2.089			
Model 8						
(Intercept)	−2.950714373	0.0031704	−17.999	0.7345	0.8333	0.9231
miR-221-3p	3.001448441	0.002686985	1.714			
Model 9						
(Intercept)	−2.56312352	0.010373514	−17.351	0.7865	0.7083	1
miR-100-5p	2.619759598	0.008799177	1.264			
Model 10						
(Intercept)	−3.185391733	0.001445582	−9.164	0.2886	1	0.6923
miR-485-3p	3.350834925	0.000805683	0.57			

## Data Availability

The data presented in this study are available in this article and Supplementary Materials.

## Supplementary Materials

The following supporting information can be downloaded at: https://www.mdpi.com/article/10.3390/ijms26083872/s1.
